# Early Disseminated Cryptococcosis Following Orthotopic Heart Transplantation Requiring VA‐ECMO: A Case Report

**DOI:** 10.1155/crcc/5744015

**Published:** 2026-09-08

**Authors:** Vanessa Petrich, Lucas Deschenes

**Affiliations:** ^1^ University of Minnesota Medical School, Minneapolis, Minnesota, USA, umn.edu; ^2^ Department of Anesthesiology, University of Minnesota Twin Cities, Minneapolis, Minnesota, USA, umn.edu

## Abstract

Disseminated cryptococcosis is a rare but serious opportunistic infection following solid organ transplantation and rarely occurs within the first month of transplantation. We report a case of disseminated *Cryptococcus neoformans* infection occurring 14 days after orthotopic heart transplantation which required mechanical circulatory support with venoarterial extracorporeal membrane oxygenation (VA‐ECMO). Management was complicated by alterations in antifungal pharmacokinetics and the inability to perform serial lumbar punctures while anticoagulated. Antifungal therapy with nonliposomal amphotericin B, flucytosine, and fluconazole was administered. A combination of optic nerve sheath diameter measurements, CT scans, and evaluation for papilledema was used as temporizing, noninvasive surrogates for intracranial pressure monitoring until lumbar punctures could be safely performed. The patient was successfully decannulated, weaned from mechanical support, and discharged from the intensive care unit.

## 1. Introduction

Disseminated cryptococcal infection occurs in up to 5% of solid organ transplant recipients, with the highest incidence occurring after heart transplants [1, 2]. Acute presentations within 30 days of transplantation remain uncommon. We report a case of early disseminated cryptococcosis occurring 14 days following orthotopic heart transplantation complicated by right ventricular failure requiring venoarterial extracorporeal membrane oxygenation (VA‐ECMO), which posed significant diagnostic and therapeutic challenges.

## 2. Case Description

A 44‐year‐old man with a history of HFrEF secondary to mixed ischemic/nonischemic cardiomyopathy and chronic kidney disease underwent orthotopic heart transplantation after a prolonged hospitalization requiring escalating cardiovascular support. Immunosuppressive induction was with basiliximab and corticosteroids, whereas maintenance immunosuppression was with tacrolimus, mycophenolate, and prednisone. His immediate postoperative course was uneventful; however, on postoperative Day 9, he developed subjective chills and leukocytosis. On postoperative Day 14, his clinical status deteriorated with cardiogenic shock secondary to right ventricular dysfunction requiring inotropic support, acute hypoxic respiratory failure resulting in intubation and mechanical ventilation, and ultimately initiation of VA‐ECMO.

Blood cultures obtained subsequently grew *Cryptococcus neoformans*, with a markedly elevated serum cryptococcal antigen titer of 1:81,920. Chest CT demonstrated bilateral upper lobe nodular opacities. Lumbar puncture, obtained on POD 21, revealed an opening pressure of 24 cmH_2_0, lymphocytic pleocytosis, and a CSF cryptococcal antigen titer of 1:40. In light of these positive titers, his immunosuppressive regimen was modified with a dose reduction of tacrolimus and discontinuation of mycophenolate. Antifungal therapy was initiated with flucytosine and amphotericin B, and fluconazole was added as a third agent. Treatment with triple therapy occurred for 14 days, followed by an additional 14 days of amphotericin B and fluconazole, followed by a prolonged course of fluconazole monotherapy. Tacrolimus levels were checked regularly with a goal trough level of 10–15 ng/mL.

**Figure 1:**
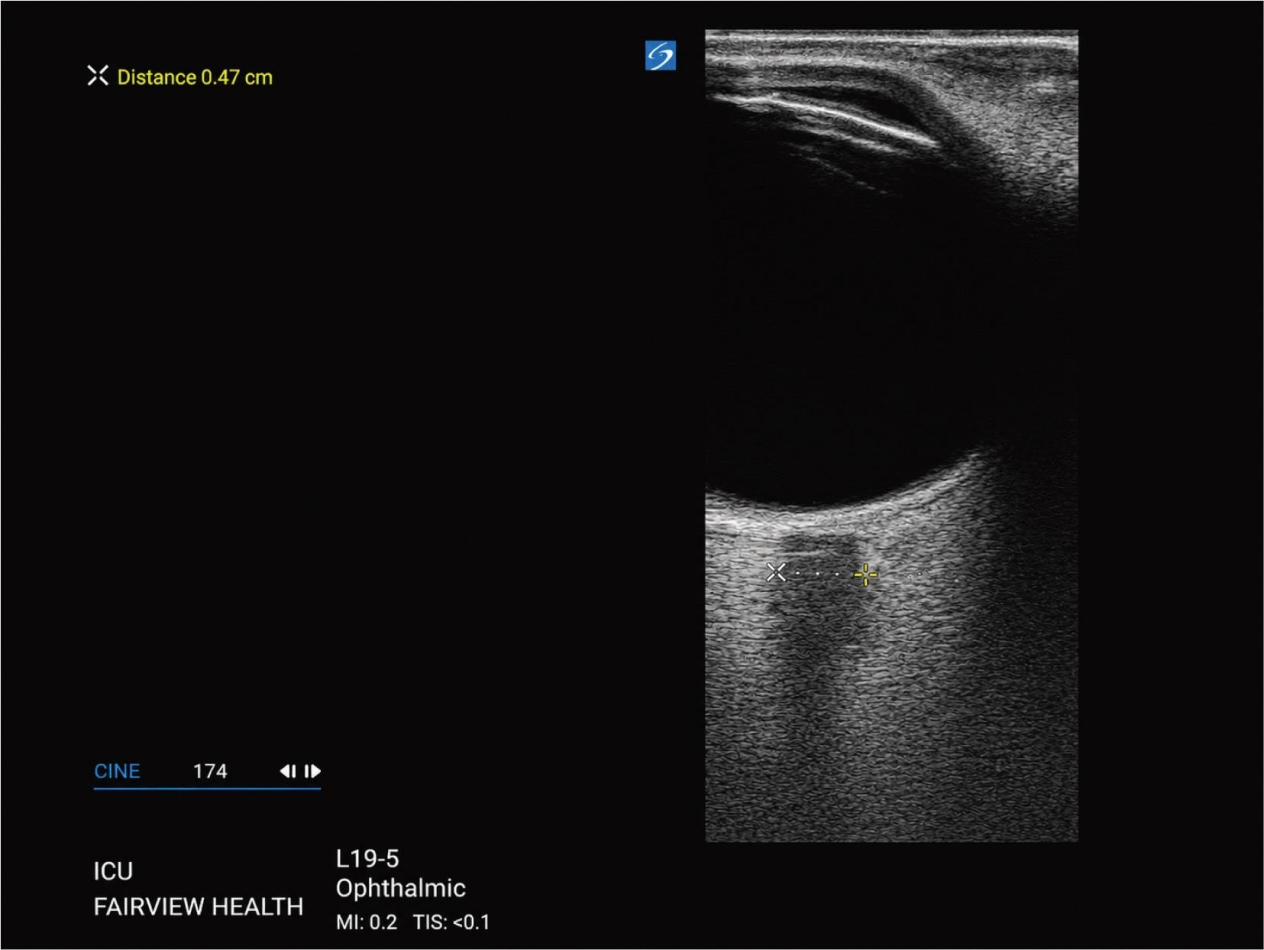
Bedside ocular ultrasonography demonstrating an optic nerve sheath diameter of 0.47 cm within normal limits and not suggestive of elevated intracranial pressure. AI was used in an attempt to decrease glare and increase image quality.

**Figure 2:**
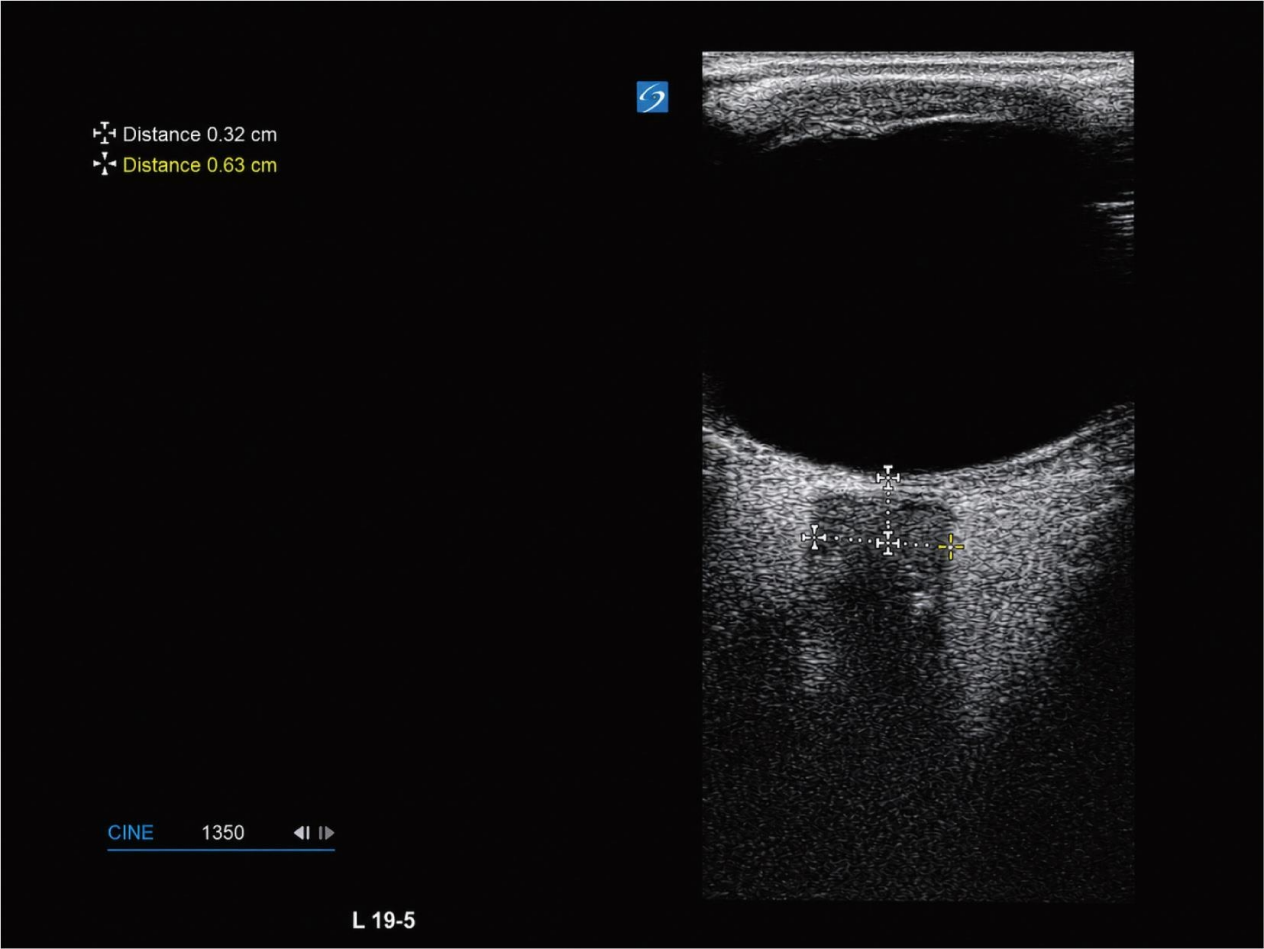
Bedside ocular ultrasonography demonstrating an optic nerve sheath diameter of 0.63 cm concerning for increased intracranial pressure. AI was used in an attempt to decrease glare and increase image quality.

The patient was intermittently encephalopathic following clinical deterioration; however, this was short lived and recovered to baseline after stabilization. There were no seizures or other clinical neurological findings suggestive of increased intracranial pressure; however, examination was limited by sedation and mechanical ventilation. CT scans were without evidence of increased ICP and an initial eye exam was without papilledema. Daily optic nerve sheath diameter measurements were monitored as a surrogate for ICP in the setting of systemic anticoagulation required for VA‐ECMO. In our patient, as optic nerve sheath diameter measurements approached and exceeded the upper limits of normal (Figures 1 and 2, respectively), heparin was temporarily held to facilitate lumbar puncture while on VA‐ECMO.

On POD 24, the patient was transitioned to a percutaneous right ventricular assist device (RVAD) with oxygenator and decannulated from VA‐ECMO, following which lumbar punctures were resumed which demonstrated persistently high opening pressures necessitating continued intermittent drainage. The oxygenator was discontinued on POD 28. The patient was extubated to high flow nasal cannula with inhaled nitric oxide on POD 29, and the RVAD was removed on POD 34. He was transferred out of the ICU on POD 44 and ultimately discharged on POD 101 after a prolonged hospital course.

He is currently on maintenance therapy of daily fluconazole dosed at 200 mg, which he will continue indefinitely. Both serum and CSF cryptococcal antigens have declined and stabilized around 1:2560 and 1:5, respectively. He has no apparent neurological sequelae. Although his disseminated cryptococcal infection has been well controlled, he was found to have a 2R rejection of his graft on endomyocardial biopsy secondary to nonadherence to his immunosuppressive medication regimen. This is currently being closely treated and monitored with frequent echocardiography and cardiac catheterizations.

## 3. Discussion

This case highlights several key novel and unique considerations. Firstly, the unusually early onset of disseminated cryptococcosis—within 2 weeks of transplantation—underscores the importance of maintaining a high index of suspicion even in the immediate postoperative period. The extremely early onset naturally raises concern for donor‐derived disease; however, other recipients tested negative for cryptococcal antigen.

Secondly, it is important to discuss the unique medication modifications and monitoring that occurred with this case. Firstly, ECMO introduced substantial pharmacokinetic variability through increased volume of distribution, circuit sequestration of lipophilic and protein‐bound drugs, and altered clearance [3]. These factors complicate antifungal therapy, particularly for amphotericin B. We opted to use nonliposomal amphotericin B dosed at 1 mg/kg/day to minimize the sequestration of drug in the ECMO circuit, potentially resulting in subtherapeutic dosing. Secondly, fluconazole can mediate supratherapeutic levels of both corticosteroids and tacrolimus through CYP3A4 inhibition. This interaction is likewise complicated by the many external factors that affect CYP3A4 metabolism in critically ill patients [4]. The commonly cited approach is to reduce tacrolimus dosing by 50%–60% at the initiation of fluconazole [5]. Tacrolimus levels should be checked regularly after initiation of fluconazole and then spot checked once stable with a goal to maintain a trough level of 10–15 ng/mL in the first 3 months following transplant.

Thirdly, management of intracranial hypertension required deviation from standard practice. Optic nerve sheath diameter measurement offers a noninvasive surrogate for ICP with high sensitivity in other clinical contexts; however, its role in cryptococcal meningitis remains uncertain [6]. In this case, optic nerve sheath monitoring was used as a temporizing strategy until lumbar punctures could safely be performed. This hybrid approach of optic nerve sheath measurements, papilledema exams and CT scans balanced the risks of invasive procedures during ECMO against the known increased mortality associated with untreated intracranial hypertension. The pathophysiology behind elevated ICP in cryptococcal meningitis differs from other causes of elevated intracranial pressure [7]. Because optic nerve sheath diameter measurements have not been validated as a tool for monitoring intracranial pressure in patients with disseminated cryptococcosis, we resumed intermittent lumbar punctures following decannulation from VA‐ECMO as per IDSA guidelines [8].

Few reports have described disseminated cryptococcosis requiring mechanical circulatory support, and we found no reports describing disseminated cryptococcosis occurring within 2 weeks of orthotopic heart transplantation necessitating hemodynamic support with VA‐ECMO. This case highlights a convergence of challenges not previously described in the literature, notable for the use of optic nerve sheath diameter measurements as a temporizing, noninvasive strategy to monitor intracranial pressure when lumbar puncture was not feasible, as well as the implementation of triple antifungal therapy to address concerns of altered pharmacokinetics during ECMO. These adaptations underscore the need for flexible, multidisciplinary approaches in managing severe opportunistic infections in critically ill transplant recipients, particularly when standard diagnostic and therapeutic pathways are constrained.

## Funding

No funding was received for this manuscript.

## Disclosure

All authors have read and approved the final version of the manuscript. Dr. Lucas Deschenes, M.D., had full access to all of the data in this study and takes complete responsibility for the integrity of the data and the accuracy of the data analysis. Ms. Vanessa Petrich affirms that this manuscript is an honest, accurate, and transparent account of the study being reported, that no important aspects of the study have been omitted, and that any discrepancies from the study as planned have been explained.

## Consent

Informed consent was obtained from the patient′s next of kin for publication of this case report and any accompanying images.

## Conflicts of Interest

The authors declare no conflicts of interest.

## Data Availability

Data sharing is not applicable to this article as no datasets were generated or analyzed during the current study.
